# Co-Expression Effect of SLC7A5/SLC3A2 to Predict Response to Endocrine Therapy in Oestrogen-Receptor-Positive Breast Cancer

**DOI:** 10.3390/ijms21041407

**Published:** 2020-02-19

**Authors:** Lutfi H. Alfarsi, Rokaya El-Ansari, Madeleine L. Craze, Brendah K. Masisi, Omar J. Mohammed, Ian O. Ellis, Emad A. Rakha, Andrew R. Green

**Affiliations:** 1 Nottingham Breast Cancer Research Centre, Division of Cancer and Stem Cells, School of Medicine, University of Nottingham Biodiscovery Institute, University Park, Nottingham NG7 2RD, UK; lutfi.alfarsi@nottingham.ac.uk (L.H.A.); msxre2@exmail.nottingham.ac.uk (R.E.-A.); mszmc@exmail.nottingham.ac.uk (M.L.C.); msxbkma@exmail.nottingham.ac.uk (B.K.M.); msaom1@exmail.nottingham.ac.uk (O.J.M.); mrzie@exmail.nottingham.ac.uk (I.O.E.); mrzear1@exmail.nottingham.ac.uk (E.A.R.); 2 Cellular Pathology, Nottingham University Hospitals NHS Trust, Nottingham City Hospital, Hucknall Road, Nottingham NG5 1PB, UK

**Keywords:** breast cancer, oestrogen receptor, endocrine resistance, SLC7A5, SLC3A2

## Abstract

The majority of breast cancers are oestrogen-receptor-positive (ER+) and are subject to endocrine therapy; however, an unpredictable subgroup of patients will develop resistance to endocrine therapy. The SLC7A5/SLC3A2 complex is a major route for the transport of large neutral essential amino acids through the plasma membrane. Alterations in the expression and function of those amino-acid transporters lead to metabolic reprogramming, which contributes to the tumorigenesis and drug resistance. This study aims to assess the effects and roles of SLC7A5/SLC3A2 co-expression in predicting responses to endocrine therapy in patients with ER+ breast cancer. The biological and clinical impact of SLC7A5/SLC3A2 co-expression was assessed in large annotated cohorts of ER+/HER2− breast cancer with long-term follow-up at the mRNA and protein levels. In vitro experiments were conducted to investigate the effect of SLC7A5/SLC3A2 knockdown in the proliferation of cancer cells and to the sensitivity to tamoxifen. We found that proliferation-related genes are highly expressed in a subgroup of patients with high SLC7A5/SLC3A2, and knockdown of SLC7A5/SLC3A2 decreased proliferation of ER+ breast cancer cells. In patients treated with endocrine therapy, high SLC7A5/SLC3A2 co-expression was associated with poor patient outcome, and depletion of SLC7A5/SLC3A2 using siRNA increased the sensitivity of breast cancer cells to tamoxifen. On the basis of our findings, SLC7A5/SLC3A2 co-expression has the potential of identifying a subgroup of ER+/HER2− breast cancer patients who fail to benefit from endocrine therapy and could guide the choice of other alternative therapies.

## 1. Introduction

Endocrine therapy is the major treatment for oestrogen-receptor-positive (ER+) breast cancer, and the measurement of hormone-receptor protein expression, using immunohistochemistry, is essential, but not sufficient to accurately predict benefit from endocrine treatment. However, oestrogen-independent growth often exists de novo at diagnosis or develops during the course of endocrine therapy. Therefore, additional predictive biomarkers of the benefits from endocrine therapy in ER+ breast cancer are still essential [1].

Metabolic reprogramming is one of the hallmarks of cancer, and recently has attracted great attention because it may reveal clinical significance as predictive markers and therapeutic targets. Amino-acid transporters are transmembrane proteins that have essential functions in protein synthesis to maintain cell integrity and cell cycle progression. Additionally, they play vital roles in cancer growth by regulating energy metabolism, gene expression and signalling pathways [2]. In order to fuel rapid proliferation, cancer cells display an increased demand for amino-acid transporters. Alterations in the expression and function of those transporters lead to metabolic reprogramming, which changes intracellular amino-acid levels, contributing to the tumorigenesis [3].

SLC7A5, also known as LAT1, is a sodium-independent transporter that supplies cells with large neutral amino acids, which are not only required for protein synthesis but also contribute to various signalling pathways. SLC7A5 forms a heteromeric amino-acid transporter complex with SLC3A2, also known as 4F2hc or CD98, to stabilize and facilitate its translocation to the plasma membrane [4,5]. In addition to the potential role of SLC3A2 in integrin signalling [6,7], a recent study reveals that SLC3A2 is essential for the transport activity of the SLC7A5/SLC3A2 complex [8]. The expression of the SLC3A2/SLC7A5 complex is co-dependent [9], and knockdown of either reduces transport of glutamine or leucine, which causes a marked reduction in cell size [10].

Despite several studies that have shown the prognostic role of SLC7A5 or SLC3A2 in cancers [11,12,13,14,15,16,17,18], the relation of their co-expression with endocrine therapy efficacy in ER+/HER2− breast cancer has yet to be reported. In this study, we aim to evaluate the predictive value of the co-expression of the SLC7A5/SLC3A2 complex as a clinical marker of benefit from endocrine therapy in early ER+ breast cancer.

## 2. Results

### 2.1. SLC7A5/SLC3A2 Co-Expression and Clinicopathological Characteristics

**Figure 1:**
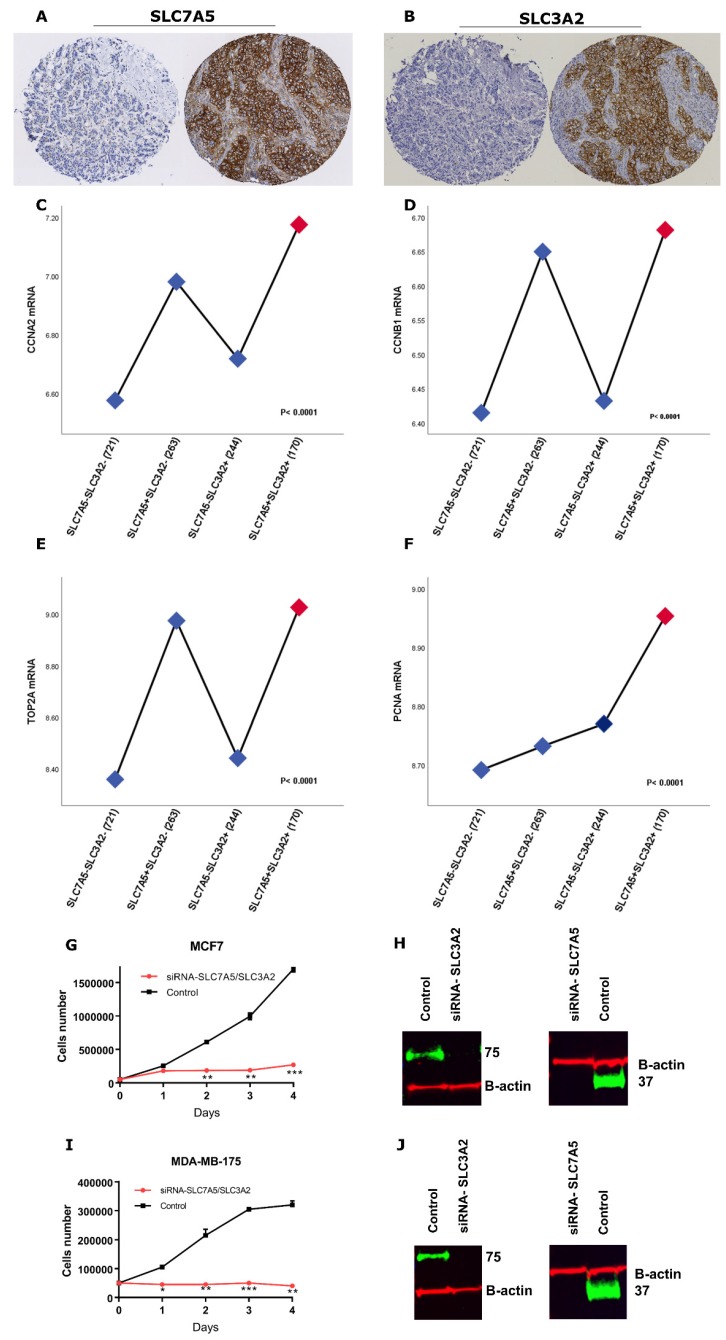
(**A**) Negative and positive SLC7A5 protein expression and (**B**) negative and positive SLC3A2 protein expression in invasive breast cancer cores using IHC. Association of *SLC7A5/SLC3A2 mRNA* co-expression with proliferation associated-genes, including (**C**) *CCNA2*, (**D**) *CCNB1*, (**E**) *TOP2A* and (**F**) *PCNA*, using the METABRIC cohort. A one-way ANOVA with the post-hoc Tukey multiple comparison test was used, and *p* values of ≤ 0.05 were considered significant. (**G**) MCF-7 and (**I**) MDA-MB-175-VII cells were transfected with control siRNA or SLC7A5 and SLC3A2 siRNA (si-SLC7A5/SLC3A2) for 24 h. Cells were then seeded in duplicate in corresponding media, and on the indicated days, cells were trypsinized and stained with trypan-blue and counted. Results shown are mean ± SE of two independent experiments. Asterisks denote *p*-values as follows: * *p* < 0.05; ** *p* < 0.005; *** *p* < 0.001. The efficiency of SLC7A5 and SLC3A2 knockdown in (**H**) MCF7 and (**J**) MDA-MB-175-VII cells were confirmed by Western blotting.

Protein expression of the two amino-acid transporters was predominantly in the membrane of the invasive breast cancer cells, with intensity levels varying from absent to high (Figure 1A,B). Both SLC7A5 and SLC3A2 protein expression were dichotomized into low and high using a modified histochemical score (H-score) of 15. To explore the prognostic and predictive value of SLC7A5/SLC3A2 co-expression in ER+/HER2− breast cancer at the *mRNA* and protein levels, the cases were divided into four categories (SLC7A5−SLC3A2−, SLC7A5+SLC3A2−, SLC7A5−SLC3A2+ and SLC7A5+SLC3A2+).

To determine the association of clinicopathological factors with *mRNA SLC7A5/SLC3A2* co-expression in ER+/HER2− breast cancer, the Molecular Taxonomy of Breast Cancer International Consortium (METABRIC) cohort was used. Results showed that tumours with *SLC7A5+/SLC3A2+ mRNA* co-expression were positively associated with high grade tumours, a poor Nottingham Prognostic Index (NPI) and vascular invasion (*p* < 0.05), as seen in Table 1. The analysis for protein expression showed consistency with *mRNA* results, where the SLC7A5+SLC3A2+ subgroup of the patients was significantly associated with aggressive clinicopathological characteristics (*p* < 0.05; Table 1).

### 2.2. SLC7A5/SLC3A2 Co-Expression Associates with Proliferation in ER+ Breast Cancer Cells

Due to the significant association of the SLC7A5+SLC3A2+ subgroup of patients with a high rate of mitotic activity (*p* < 0.0001), we next investigated if SLC7A5/SLC3A2 co-expression is associated with proliferation of ER+ breast cancer. We used the METABRIC dataset to investigate the association between the *SLC7A5/SLC3A2 mRNA* subgroups and the expression of well-characterized proliferation related-genes. We found that the *SLC7A5+SLC3A2+* subgroup of patients associate with a high expression of *CCNA2* (cell cycle progression regulator), *CCNB1* (G2/M transition phase regulator), *TOP2A* (controls/alters topologic state of DNA during transcription) and *PCNA* (eukaryotic DNA replication regulator) (*p* < 0.05; Figure 1C–F).

Furthermore, we transfected both MDA-MB-175-VII and MCF7 cells with siRNA targeting both SLC7A5 and SLC3A2 and determined cell proliferation. Double knockdown of these solute carriers impaired the proliferation of both MDA-MB-175-VII and MCF7 cells in compared to control siRNA transfected cells (Figure 1G–J).

### 2.3. Prognostic Value of SLC7A5/SLC3A2 Co-Expression

**Figure 2:**
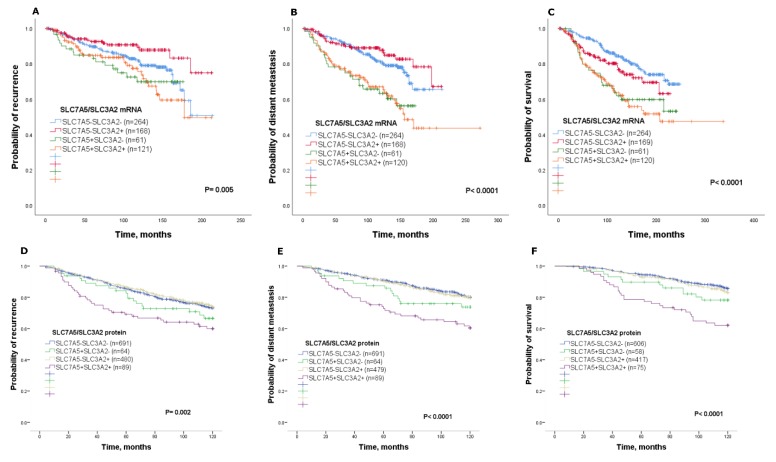
Kaplan–Meier of *SLC7A5/SLC3A2 mRNA* co-expression and patient outcome in ER+/HER2− breast cancer using the METABRIC cohort: (**A**) recurrence, (**B**) distant metastasis and (**C**) breast-cancer-specific survival. Kaplan–Meier of SLC7A5/SLC3A2 protein co-expression and patient outcome in ER+/HER2− breast cancer: (**D**) recurrence, (**E**) distant metastasis and (**F**) breast-cancer-specific survival.

Co-expression of *SLC7A5/SLC3A2 mRNA* within the METABRIC cohort was used to determine the prognostic value in ER+/HER2− breast cancer, whereby the *SLC7A5+SLC3A2+* subgroup was associated with poor clinical outcome. Specifically, patients with *SLC7A5+SLC3A2+* tumours were significantly associated with disease recurrence, distant metastasis and a high risk of death from breast cancer (*p* < 0.05; Figure 2A–C) compared with the other subgroups, which showed better clinical outcome. A Kaplan–Meier survival analysis was performed to determine patient outcome at the protein levels for SLC7A5/SLC3A2 co-expression. Results were consistent with *SLC7A5/SLC3A2 mRNA* expression whereby patients with SLC7A5+SLC3A2+ tumours had a worse outcome compared to patients of other subgroups (Figure 2D–F). In univariate analysis, SLC7A5+SLC3A2+ co-expression was a predictor of high risk for disease recurrence, distant metastasis and short survival (*p* < 0.01) compared with each of the other subgroups, as seen in Table 2.

Cox regression analysis was used to investigate the independent prognostic value of SLC7A5/SLC3A2 co-expression in ER+/HER2− patients. Results from the METABRIC cohort demonstrated that *SLC7A5+SLC3A2+* co-expression was a predictor of poor recurrence, distant metastasis and short survival (*p* = 0.05; Table 3). This was consistent when analysing protein expression, where SLC7A5+SLC3A2+ co-expression was shown to predict a high risk of distant metastasis and death from breast cancer (*p* < 0.05), but not recurrence (*p* = 0.25; Table 3). Altogether, these findings indicate that high SLC7A5/SLC3A2 co-expression associates with aggressive features in ER+/HER2− breast cancer, leading to cancer progression and short survival.

### 2.4. Predictive Value of SLC7A5/SLC3A2 Co-Expression for Endocrine Treatment Benefit

**Figure 3:**
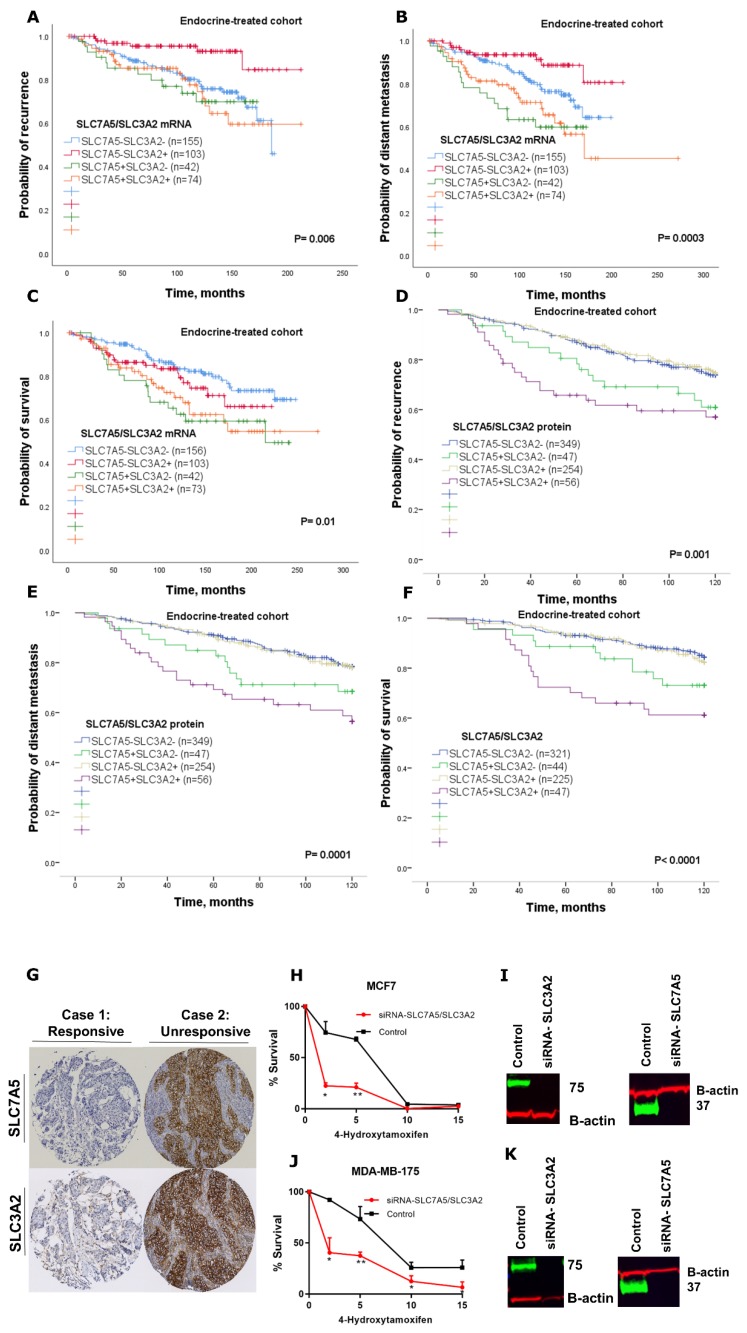
Kaplan–Meier of *SLC7A5/SLC3A2 mRNA* co-expression in patients with ER+/HER2− breast cancer who received endocrine treatment only using the METABRIC cohort: (**A**) recurrence, (**B**) distant metastasis and (**C**) breast-cancer-specific survival. Kaplan–Meier of SLC7A5/SLC3A2 protein co-expression in patients with ER+/HER2− breast cancer who received endocrine treatment only: (**D**) recurrence, (**E**) distant metastasis and (**F**) breast-cancer-specific survival. (**G**) IHC staining of SLC7A5 and SLC3A2 from two cases of patients who were subject to endocrine therapy alone. Cell viability assay in (**H**) MCF7 and (**J**) MDA-MB-175-VII cells transfected with siRNA control or siRNA targeting SLC7A5 and SLC3A2 (si-SLC7A5/SLC3A2). Cells were treated with different concentrations of 4-Hydroxytamoxifen (2, 5, 10 and 15 μM) for 72 h. Results shown are mean ± SE of at least two independent experiments performed in triplicate. Asterisks denote *p*-values as follows: * *p* < 0.05; ** *p* < 0.005. The efficiency of SLC7A5 and SLC3A2 knockdown in (**I**) MCF7 cells and (**K**) MDA-MB-175-VII was confirmed by Western blotting.

After showing that SLC7A5/SLC3A2 co-expression was associated with a worse outcome in patients with ER+ breast cancer, we next asked whether SLC7A5/SLC3A2 co-expression correlates with endocrine sensitivity. To test this, we analysed their *mRNA* and protein co-expression with clinical outcome in a subgroup of patients who received endocrine therapy alone. First, we used the METABRIC cohort to investigate the possible correlation between *mRNA* and clinical outcome; this analysis revealed that patients with *SLC7A5+SLC3A2+* tumours had a significantly worse recurrence, distant metastasis and shorter survival (*p* = 0.01; Figure 3A–C) compared to other subgroups.

To directly interrogate this association at the protein level, we performed Kaplan–Meier survival analyses for the immunohistochemistry (IHC) stained cases. First, we investigated whether singular expression of either SLC7A5 or SLC3A2 may help to identify patients who have relapsed after receiving endocrine therapy. There was no significant difference in risk of recurrence or distant metastasis between patients with low vs. high SLC7A5 or SLC3A2 expression in the endocrine-treated cohort (Supplementary Figure S1A–D). However, results of co-expression were consistent with the *mRNA* whereby patients who were subject to endocrine treatment alone and with high SLC7A5+SLC3A2+ co-expression had an adverse outcome compared to patients with other subgroups who had a better clinical outcome (*p* < 0.01; Figure 3D–F). Further, we showed representative images of IHC staining for SLC7A5 and SLC3A2 from two cases of patients who were subject to endocrine therapy alone (Figure 3G). The result demonstrates those patients whose response to endocrine treatment had a low expression of both SLC7A5 and SLC3A2, while patients who had relapsed after receiving endocrine therapy shows a high expression of those amino-acid transporters. Further analysis of Cox regression in the subgroup of patients who were treated with endocrine therapy alone revealed that SLC7A5+SLC3A2+ co-expression at both the *mRNA* and protein levels is a predictive marker for a high risk of disease recurrence, distant metastasis and breast cancer death (*p* < 0.05; Table 4).

To further investigate whether SLC7A5/SLC3A2 co-expression affects the response of ER+ breast cancer cells to tamoxifen, we knocked down SLC7A5 and SLC3A2 in combinations in MCF7 and MDA-MB-175-VII cells using siRNAs. We then treated siRNA targeted and scrambled control cells with various concentrations of 4-hydroxytamoxifen and measured cell viability. Results show 4-hydroxytamoxifen had a much greater effect on the SLC7A5/SLC3A2 siRNA target, which reduced the viability of the MCF7 and MDA-MB-175-VII cells in a concentration-dependent manner (Figure 3H–K). This finding confirms that depletion of these amino-acid transporters enhances efficiency of tamoxifen treatment in ER+ breast cancer cells.

## 3. Discussion

Amino-acids transporters are crucial for cellular functions and survival and have essential roles in regulating cellular metabolism. Alterations in the expression and function of these transporters in cancer cells are related to tumour progression and treatment resistance [3]. SLC7A5 functions in supplying amino acids to cancer cells as well as maintaining intra-cellular leucine [19,20], and SLC3A2 is required for the functional expression of SLC7A5 in tumour cells [4].

In this study, we showed the clinicopathological significance and prognostic utility of the SLC7A5/SLC3A2 complex in predicting the benefit of endocrine treatment in patients with ER+ breast cancer. Our results indicated that high co-expression of the SLC7A5/SLC3A2 complex is associated with poor prognostic clinicopathological parameters, including a larger tumour size, higher grade, poor NPI and vascular invasion. Several previous studies have shown that SLC7A5 and SLC3A2 are prognostic markers in different types of cancer [13,14,15,16,17,18]. We also have recently demonstrated that SLC7A5 and SLC3A2 singularly are associated with poor prognosis, particularly in the luminal-B subtype of breast cancer [11,12]; however, studies addressing the clinical significance of the SLC7A5/SLC3A2 complex in ER+ breast cancer and the efficacy of endocrine treatment remain lacking. In this study, we report that high SLC7A5/SLC3A2 co-expression is associated with poor clinical outcome in ER+ breast cancer patients, while those in the other combinatorial subgroups showed a better outcome. These findings identify an association between co-expression of the SLC7A5/SLC3A2 complex and a poor prognosis in ER+ breast cancer.

Proliferating cells showed increased demand for amino acids to sustain their rapid growth, and such a supply is supported by the upregulation of amino-acid transporters [3]. In this regard, we observed that the SLC7A5+SLC3A2+ subgroup is associated with high levels of mitotic activity in patients with ER+ breast cancer. This is consistent with previous work by our group and others which demonstrated that SLC7A5 and SLC3A2 are significantly correlated with the proliferative marker Ki-67 [11,12,21]. Our results also demonstrated that cases with high SLC7A5/SLC3A2 co-expression are associated with a high expression of proliferation-related genes. Previous studies reported that increased expression of proliferation markers seem to influence the biological and clinical behaviour of the cancer cells, which supported our findings [22]. In addition, we found that silencing of both SLC7A5 and SLC3A2 by RNA interference impaired the proliferation of the ER+ cancer cells. Altogether, these findings indicate that the SLC7A5/SLC3A2 complex implicated in the proliferation of ER+ breast cancer leads to tumorigenesis and the aggressiveness phenotype.

The endocrine therapy is the primary treatment of ER+ breast cancer, which represents more than 70% of breast tumours. Although hormone treatment improves overall survival and reduces risk for relapse, an unpredictable subset of patients will relapse and die as a result of the disease [1]. Therefore, prediction of those patients that may or may not benefit from adjuvant endocrine therapy would be beneficial for ER+ breast cancer patients. Our study further establishes the impact of SLC7A5/SLC3A2 co-expression on the clinical outcome and efficacy of adjuvant endocrine treatment in a large cohort of patients with ER+ breast cancer. In our study, we have demonstrated that the individual expression of either SLC7A5 or SLC3A2 is insufficient to predict responses to endocrine therapy, but with using the co-expression, the ability to predict poor patient outcome is greatly enhanced. Thus, we found that patients with ER+ tumours expressing a high SLC7A5/SLC3A2 complex correlated with poor outcome after receiving endocrine therapy, suggesting that assessment of these solute carriers’ expression prior to adjuvant treatment could predict patients who are highly likely to fail to obtain a benefit from the endocrine therapy. Indeed, we found that knockdown of SLC7A5 and SLC3A2 expression increase the sensitivity of breast cancer cells to tamoxifen, which suggests that targeting co-expression of the SLC7A5/SLC3A2 complex might be a potential therapeutic approach to improve the efficacy of endocrine therapy in ER+/HER2− early breast cancer.

Despite our data demonstrating the prognostic and predictive utility of SLC7A5/SLC3A2 co-expression in ER+ breast cancer, the exact mechanism of how the heterodimeric complex of SLC7A5/SLC3A2 contributes to endocrine resistance is unclear and requires further mechanistic investigations. Such further investigation should use tamoxifen-resistant cell lines to demonstrate that knockdown of these genes changes the sensitivity to tamoxifen. The predictive value of SLC7A5/SLC3A2 co-expression regarding the benefit from endocrine therapy will require further investigation in clinical trials of primary breast cancer treatment.

Here we showed that high co-expression of the SLC7A5/SLC3A2 complex could be used as a prognostic marker to predict a poor response to endocrine treatment in ER+ breast cancer. We therefore propose that the co-expression of the SLC7A5/SLC3A2 complex could potentially be measured in ER+ breast cancer patients to identify those patients who will fail to benefit from endocrine therapy, potentially reducing the risk of relapse; its presence may also guide the clinician towards a rational choice of other alternative therapies for these patients.

## 4. Materials and Methods

### 4.1. Patients mRNA Expression Cohort

To investigate the prognostic value of *SLC7A5/SLC3A2 mRNA* expression in ER+/HER2− breast cancer patients and its role as predictive marker of clinical outcome for patients who were subject to endocrine treatment alone, we used the (METABRIC) cohort [23]. The characteristics of this cohort are summarized in Table 5. This study was performed according to the REMARK guidelines for tumour prognostic studies [24], and approved by the Nottingham Research Ethics Committee 2 under the title “Development of a molecular genetic classification of breast cancer” (REC202313 April 2019). This paper follows the rules of the Declaration of Helsinki.

### 4.2. Patients Protein Expression Cohort

SLC7A5 and SLC3A2 protein expression was assessed in a well-characterized series of ER+/HER2− primary invasive breast cancer patients, with long-term follow-up. Patients were presented at Nottingham City Hospital between 1989 and 2006. Patient management was uniform and based on tumour characteristics by NPI and hormone receptor status. No adjuvant therapy was given to patients with a good prognostic NPI score (≤3.4), while for patients with poor NPI scores (>3.4) endocrine therapy was given. Premenopausal patients within the moderate and poor prognostic NPI were given chemotherapy, whereas postmenopausal patients with a moderate or poor NPI were candidates for hormonal therapy. None of the patients received neoadjuvant therapy. The characteristics of this cohort are summarized in Table 5.

### 4.3. IHC Staining and Evaluation

The IHC staining was performed on 4 μm tissue microarrays sections using a Novolink polymer detection system (RE7150-K, Leica Biosystems, Newcastle, UK), as previously described [12]. Evaluation of membranous staining for SLC7A5 1:200 (EPR17573, Abcam, Cambridge, UK) and SLC3A2 1:2000 (HPA017980, Sigma-Aldrich, Dorset, UK) were based on a semi-quantitative assessment of invasive tumour cells using a modified H-score as previously described [11,12]. TMA cores were only assessed if the tumour burden was >15%.

### 4.4. Clinical Outcome and Events Definition

Clinical outcomes, including breast-cancer-specific survival, was defined as the time in months from the diagnosis to the date of death from breast cancer. Recurrence-free survival was defined as the time in months from diagnosis until developing local or regional recurrence. Distant metastasis-free survival was defined as the time in months from diagnosis until developing distant metastasis. For the benefit of endocrine therapy, the expression of SLC7A5/SLC3A2 analysis was associated with the clinical outcome on the endocrine-treated cohort only. Secondary outcomes include associations with clinical–pathological parameters. Survival was censored if the patient was still alive, lost to follow-up or died from other causes.

### 4.5. Cell Lines and Reagents

ER+ breast cancer cell lines MCF7 and MDA-MB-175-VII were obtained from the American Type Culture Collection (Rockville, MD, USA). MCF7 cells were cultured in RPMI-1640 while MDA-MB-175-VII cells were maintained in Dulbecco’s Modified Eagle medium, both supplemented with 10% foetal bovine serum. Cells were maintained in a 37 °C humidified incubator with 5% carbon dioxide. All cells were regularly tested for mycoplasma contamination. All the cell lines have been authenticated using Short Tandem Repeat (STR) profiling. Rabbit anti-SLC3A2 (HPA017980) and mouse anti-β-actin were purchased from (Sigma-Aldrich, Dorset, UK), while rabbit anti-SLC7A5 (EPR17573) was purchased from (Abcam, Cambridge, UK). The tamoxifen active metabolite 4-Hydroxytamoxifen (SML1666) was purchased from (Sigma-Aldrich, Dorset, UK). siRNAs targeting SLC7A5 (ID: s15653), SLC3A2 (ID: s12943) and scrambled negative siRNA control (ID: 4390843) were synthesized by (Ambion, ThermoFisher Scientific, Huntingdon, UK). The 3-(4,5-dimethyl-2-yl)-5-(3-carboxymethoxyphenyl)-2-(4-sulfophenyl)-2H-tetrazolium, inner salt (MTS), (CellTiter 96^®®^ AQueous One Solution Cell Proliferation Assay) was purchased from (Promega, Southampton, UK). Lipofectamine RNAiMAX was purchased from (ThermoFisher Scientific, Gloucester, UK).

### 4.6. Transient siRNA Knockdown of SLC7A5 and SLC3A2

MCF7 and MDA-MB-175-VII cells were transfected with SLC7A5 siRNA, SLC3A2 siRNA or scrambled negative control siRNA using lipofectamine RNAiMAX (Invitrogen) according to the manufacturer’s protocol. SLC7A5 and SLC3A2 siRNA knockdown efficiency was tested using Western blotting. A total of 24 h post transfection, transfected cells were trypsinized and seeded for a tamoxifen sensitivity assay and proliferation assay as detailed below.

### 4.7. Proliferation Assay

Control or transfected MCF7 and MDA-MB-175-VII cells were seeded in duplicate in corresponding media in 6-well plates at a density of 50,000 cells per well. On the indicated days, the cells were trypsinized and stained with trypan-blue and counted.

### 4.8. Tamoxifen Sensitivity Assay

Transfected MCF7 and MDA-MB-175-VII cells were seeded in triplicate in 96-well plates at density of 2000 cells per well. The following day, cells were treated with increasing concentrations of 4-Hydroxytamoxifen (2–15 μM). At 72 h post-incubation, cell viability was measured by MTS assay using Infinite F50 (Tecan, Männedorf, Switzerland).

### 4.9. Western Blotting

Cells were lysed with lysis buffer containing RIPA buffer (ThermoFisher Scientific, Gloucester, UK), mini protease inhibitor cocktail complete (Roche, Welwyn Garden City, UK) and phosphatase inhibitor (Sigma-Aldrich, Dorset, UK). Samples were loaded on 12% or 4%–12% SDS-polyacrylamide gel electrophoresis (PAGE) gels and transferred to PVDF membranes (Immobilon-FL). Membranes were blocked for 1 h with 5% Marvel milk powder in phosphate-buffered saline with 0.1% Tween-20 (PBST) and incubated overnight with the following primary antibodies: anti-SLC7A5 (1:200), anti-SLC3A2 (1:2000) or Anti-β-actin (1:5000) as a loading control. Membranes were washed three times with PBST followed by incubation for 1 h with IRDye 800CW and 600RD fluorescent secondary antibodies (1:15000; 926-32213 and 926-68072, LI-COR Biosciences, Cambridge, UK). Membranes were washed three times with PBST before the analysis of immunoblotting was performed using the Odyssey Fc with Image Studio 4.0 (LI-COR Biosciences).

### 4.10. Patient Data Analysis and Statistics

Statistical analysis was performed using SPSS 24.0 statistical software (SPSS Inc., Chicago, IL, USA). The analysis for this study compared SLC7A5−SLC3A2− (both SLC7A5 and SLC3A2 low expression), SLC7A5+SLC3A2− (high SLC7A5 and low SLC3A2 expression), SLC7A5−SLC3A2+ (low SLC7A5 and high SLC3A2 expression) and SLC7A5+SLC3A2+ (both SLC7A5 and SLC3A2 showing high expression). The chi-squared test was used to evaluate the significance association with clinicopathological parameters. For the continuous variables, differences between three or more groups were assessed using one-way ANOVA with the post-hoc Tukey multiple comparison test. Kaplan–Meier analysis were used to assess the clinical outcome. A multivariate Cox regression analysis with adjustment of covariates was used to identify the independent prognostic biomarkers. The Benjamini–Hochberg procedure for multiple test correction was performed when applicable. The dichotomization of SLC7A5 and SLC3A2 *mRNA* and protein expression into low and high groups was determined using X-Tile (X-Tile Bioinformatics Software, Yale University, version 3.6.1).

Student’s *t*-tests using PRISM were performed to determine the effects of SLC7A5 and SLC3A2 knockdown on cell proliferation and tamoxifen sensitivity. Data was expressed as the mean ± SE of three independent experiments performed in triplicate, or otherwise specified. All statistical tests were two-sided, and *p* values of ≤ 0.05 were considered significant.

## Figures and Tables

**Table 1:** Clinicopathological associations of SLC7A5/SLC3A2 co-expression in ER+/HER2− breast cancer.

METABRIC Cohort			SLC7A5/SLC3A2 mRNA			
Parameters	*SLC7A5^−^*	*SLC7A5^+^*	*SLC7A5^−^*	*SLC7A5^+^*	*p*	*p* *
	*SLC3A2^−^*	*SLC3A2^−^*	*SLC3A2^+^*	*SLC3A2^+^*		
	No. (%)	No. (%)	No. (%)	No. (%)		
**Size**					0.039	0.06
*<2 cm*	67 (72.8)	9 (9.8)	9 (9.8)	7 (7.6)		
*≥2 cm*	48 (52.7)	17 (18.7)	12 (13.2)	14 (15.4)		
**Grade**					3.8 × 10^−12^	<0.0001
*1*	52 (67.5)	2 (2.6)	17 (22.1)	6 (7.8)		
*2*	132 (51.8)	20 (7.8)	78 (30.6)	28 (9.8)		
*3*	59 (28.5)	23 (11.1)	60 (29)	65 (31.4)		
**Nodal Stage**					0.2	0.23
*1*	77 (67.5)	13 (11.4)	14 (12.3)	10 (8.8)		
*2*	34 (59.6)	10 (17.5)	4 (7)	9 (15.8)		
*3*	4 (36.4)	2 (18.2)	3 (27.3)	2 (18.2)		
*NPI*					8.3 × 10^−8^	<0.0001
*GPG*	111 (55.8)	11 (5.5)	63 (31.7)	14 (7)		
*MPG*	121 (42.3)	23 (8)	79 (27.6)	63 (22)		
*PPG*	15 (22.4)	12 (17.9)	21 (31.3)	19 (28.4)		
**Vascular invasion**					0.01	0.02
*Negative*	82 (71.3)	12 (10.4)	12 (10.4)	9 (7.8)		
*Definite*	33 (48.5)	14 (20.6)	9 (13.2)	12 (17.6)		
**PR**					0.1	0.14
*Negative*	60 (40.5)	8 (5.4)	48 (32.4)	32 (21.6)		
*Positive*	184 (45.9)	38 (9.5)	115 (28.7)	64 (16)		
**Nottingham Cohort**			**SLC7A5/SLC3A2 protein**			
**Size**					0.002	0.003
*<2 cm*	432 (54.8)	27 (3.4)	288 (36.5)	42 (5.3)		
*≥2 cm*	266 (48.6)	37 (6.8)	197 (36)	47 (8.6)		
**Grade**					2.6 × 10^−35^	<0.0001
*1*	184 (65.2)	0 (0)	96 (34)	2 (0.8)		
*2*	356 (56)	20 (3.1)	245 (38.5)	15 (2.4)		
*3*	158 (37.9)	44 (10.6)	144 (34.5)	71 (17)		
**Mitosis**					1.1 × 10^−32^	<0.0001
*1*	436 (59.4)	10 (1.4)	279 (38)	9 (1.2)		
*2*	133 (49.8)	21 (7.9)	90 (33.7)	23 (8.6)		
*3*	109 (35.5)	32 (10.4)	110 (35.8)	56 (18.2)		
**Nodal Stage**					0.136	0.15
*1*	450 (52.7)	34 (4)	316 (37)	54 (6.3)		
*2*	204 (52.7)	20 (5.2)	137 (35.4)	26 (6.7)		
*3*	44 (47.3)	10 (10.8)	31 (33.3)	8 (8.6)		
*NPI*					1.1 × 10^−16^	<0.0001
*GPG*	339 (60)	9 (1.6)	209 (37)	8 (1.4)		
*MPG*	297 (49)	36 (5.9)	214 (35.3)	59 (9.7)		
*PPG*	62 (38)	19 (11.7)	60 (36.8)	22 (13.5)		
**Vascular invasion**					0.0004	0.0009
*Negative*	500 (54.6)	30 (3.3)	329 (35.9)	57 (6.2)		
*Definite*	197 (47.1)	34 (8.1)	156 (37.3)	31 (7.4)		
**PR**					0.116	0.13
*Negative*	147 (47.7)	20 (6.5)	115 (37.3)	26 (8.4)		
*Positive*	548 (53.7)	44 (4.3)	366 (35.8)	63 (6.2)		

*p* *: Adjusted *p* value. NPI: Nottingham prognostic index; GPG: Good prognostic group; MPG: Moderate prognostic group; PPG: Poor prognostic group. PR: Progesterone Receptor.

**Table 2:** Univariate analysis of associations between SLC7A5/SLC3A2 co-expression in ER+/HER2− breast cancer of the Nottingham cohort.

SLC7A5−SLC3A2− vs	Outcome	Hazard Ratio	95% Confidence Interval	*p*	*p* *
SLC7A5+SLC3A2−	Recurrence	1.3	0.8–2.0	0.239	0.4
	Distant metastasis	1.4	0.8–2.4	0.147	0.2
	Survival	1.6	0.9–3.0	0.106	0.2
SLC7A5−SLC3A2+	Recurrence	0.9	0.7–1.2	0.720	0.9
	Distant metastasis	1.0	0.7–1.3	0.821	1.0
	Survival	1.1	0.8–1.6	0.289	0.3
SLC7A5+SLC3A2+	Recurrence	1.7	1.2–2.5	0.002	0.008
	Distant metastasis	2.4	1.6–3.5	0.000004	<0.0001
	Survival	3.2	2.0–4.9	1.0 × 10^−7^	<0.0001

*p* *: Adjusted *p* value.

**Table 3:** Multivariate cox analysis of associations between SLC7A5/SLC3A2 co-expression and clinicopathological parameters in the ER+/HER2− cohort.

***mRNA* Expression**			
	**Recurrence-free survival**		
	HR (95% CI)	*p*	*p* *
SLC7A5−SLC3A2− vs. SLC7A5+SLC3A2+	2.0 (1.1–3.7)	0.01	0.05
Tumour size	1.2 (0.8–2.0)	0.2	0.3
Tumour grade	1.3 (0.9–1.9)	0.09	0.2
Nodal stage	1.1 (0.7–1.5)	0.6	0.7
	**Distant metastasis-free survival**		
	HR (95% CI)	*p*	*p* *
SLC7A5−SLC3A2− vs. SLC7A5+SLC3A2+	2.1 (1.1–3.9)	0.01	0.05
Tumour size	1.8 (1.0–3.1)	0.02	0.05
Tumour grade	1.5 (1.0–2.2)	0.03	0.5
Nodal stage	1.3 (0.9–1.9)	0.1	0.12
	**Breast-cancer-specific survival**		
	HR (95% CI)	*p*	*p* *
SLC7A5−SLC3A2− vs. SLC7A5+SLC3A2+	2.2 (1.1–4.3)	0.02	0.05
Tumour size	1.8 (1.0–3.2)	0.04	0.06
Tumour grade	2.0 (1.2–3.2)	0.002	0.01
Nodal stage	1.3 (0.8–2.0)	0.1	0.12
**Protein expression**			
	**Recurrence-free survival**		
	HR (95% CI)	*p*	*p* *
SLC7A5−SLC3A2− vs. SLC7A5+SLC3A2+	1.5 (1.0–2.4)	0.2	0.25
Tumour size	1.5 (1.1–2.0)	0.006	0.01
Tumour grade	1.4 (1.1–1.8)	0.001	0.002
Nodal stage	1.6 (1.3–1.9)	0.000008	<0.0001
	**Distant metastasis-free survival**		
	HR (95% CI)	*p*	*p* *
SLC7A5−SLC3A2− vs. SLC7A5+SLC3A2+	1.5 (1.0–2.3)	0.03	0.037
Tumour size	1.6 (1.1–2.3)	0.004	0.006
Tumour grade	1.7 (1.3–2.3)	0.00001	<0.0001
Nodal stage	1.8 (1.4–2.2)	3.5 × 10^−7^	<0.0001
	**Breast-cancer-specific survival**		
	HR (95% CI)	*p*	*p* *
SLC7A5−SLC3A2− vs. SLC7A5+SLC3A2+	1.7 (1.0–2.7)	0.02	0.03
Tumour size	1.3 (0.8–2.0)	0.16	0.2
Tumour grade	2.3 (1.7–3.3)	3.7 × 10^−7^	<0.0001
Nodal stage	1.8 (1.3–2.3)	0.00002	0.0001

*p* *: Adjusted *p* value.

**Table 4:** Multivariate cox analysis of associations between SLC7A5/SLC3A2 co-expression and clinicopathological parameters in the endocrine-treated cohort.

***mRNA* Expression**			
	**Recurrence-free survival**		
	HR (95% CI)	*p*	*p* *
SLC7A5−SLC3A2− vs. SLC7A5+SLC3A2+	1.7 (1.0–2.8)	0.02	0.03
Tumour size	1.3 (0.8–2.0)	0.17	0.2
Tumour grade	2.1 (1.4–3.0)	0.00004	0.0001
Nodal stage	1.9 (1.4–2.5)	0.000003	<0.0001
	**Distant metastasis-free survival**		
	HR (95% CI)	*p*	*p* *
SLC7A5−SLC3A2− vs. SLC7A5+SLC3A2+	1.9 (1.1–3.2)	0.008	0.01
Tumour size	1.2 (0.8–2.0)	0.2	0.25
Tumour grade	2.6 (1.7–4.0)	0.000002	<0.0001
Nodal stage	2.1 (1.6–2.9)	4.7 × 10^−7^	<0.0001
	**Breast-cancer-specific survival**		
	HR (95% CI)	*p*	*p* *
SLC7A5−SLC3A2− vs. SLC7A5+SLC3A2+	2.0 (1.1–3.6)	0.01	0.016
Tumour size	0.9 (0.5–1.6)	0.8	1
Tumour grade	3.6 (2.1–6.2)	0.000002	<0.0001
Nodal stage	2.1 (1.5–3.0)	0.00002	0.0001
**Protein Expression**			
	**Recurrence-free survival**		
	HR (95% CI)	*p*	*p* *
SLC7A5−SLC3A2− vs. SLC7A5+SLC3A2+	1.7 (1.0–2.8)	0.02	0.03
Tumour size	1.3 (0.8–2.0)	0.17	0.2
Tumour grade	2.1 (1.4–3.0)	0.00004	0.0001
Nodal stage	1.9 (1.4–2.5)	0.000003	<0.0001
	**Distant metastasis-free survival**		
	HR (95% CI)	*p*	*p* *
SLC7A5−SLC3A2− vs. SLC7A5+SLC3A2+	1.9 (1.1–3.2)	0.008	0.01
Tumour size	1.2 (0.8–2.0)	0.2	0.25
Tumour grade	2.6 (1.7–4.0)	0.000002	<0.0001
Nodal stage	2.1 (1.6–2.9)	4.7 × 10^−7^	<0.0001
	**Breast-cancer-specific survival**		
	HR (95% CI)	*p*	*p* *
SLC7A5−SLC3A2− vs. SLC7A5+SLC3A2+	2.0 (1.1–3.6)	0.01	0.016
Tumour size	0.9 (0.5–1.6)	0.8	1
Tumour grade	3.6 (2.1–6.2)	0.000002	<0.0001
Nodal stage	2.1 (1.5–3.0)	0.00002	0.0001

*p* *: Adjusted *p* value.

**Table 5:** Clinicopathological characteristics of ER+/HER2− breast cancer cohorts.

Parameters	METABRIC Cohort	Nottingham Cohort
	*mRNA*	Protein
	No. (%)	No. (%)
*Age*		
*<50*	228 (15)	370 (27.7)
*≥50*	1278 (85)	967 (72.3)
*Tumour size (cm)*		
*<2 cm*	475 (31.5)	789 (59.1)
*≥2 cm*	1031 (68.5)	547 (40.9)
*Grade*		
*1*	166 (11.5)	110 (8.4)
*2*	707 (49.1)	493 (37.6)
*3*	565 (38.4)	707 (54.0)
*Nottingham Prognostic Index*		
*GPG*	623 (41.3)	565 (42.4)
*MPG*	772 (51.2)	606 (45.4)
*PPG*	111 (7.5)	163 (12.2)
*Nodal stage*		
*1*	404 (36.2)	854 (64.0)
*2*	634 (56.8)	387 (29.0)
*3*	78 (7)	93 (7.0)
*Mitosis*		
*1*		734 (56.1)
*2*	N/A	267 (20.4)
*3*		307 (23.5)
*Vascular invasion*		
*Negative*	115 (62.8)	916 (68.5)
*Positive*	68 (37.2)	418 (31.2)
*Endocrine therapy alone*		
*No*	234 (15.5)	505 (37.7)
*Yes*	384 (25.5)	716 (53.5)
*Other* *	888 (59)	117 (8.8)
*Progesterone receptor*		
*Negative*	486 (23.2)	308 (23.2)
*Positive*	1020 (76.8)	1021 (76.8)

GPG: Good prognostic group; MPG: Moderate prognostic group; PPG: Poor prognostic group. * Include patients who received chemotherapy alone or combination of chemotherapy and endocrine therapy.

## Supplementary Materials

Supplementary materials can be found at https://www.mdpi.com/1422-0067/21/4/1407/s1.

## Abbreviations

ER+	Oestrogen Receptor Positive
NPI	Nottingham Prognostic Index
IHC	Immunohistochemistry
METABRIC	Molecular Taxonomy of Breast Cancer International Consortium
H-score	Histochemical score
