# Supplementary figures and images for: Co-Expression Effect of SLC7A5/SLC3A2 to Predict Response to Endocrine Therapy in Oestrogen-Receptor-Positive Breast Cancer
Source: Int J Mol Sci. 2020 Feb 19;21(4):1407. doi: 10.3390/ijms21041407 (PMC7073058; doi:10.3390/ijms21041407)

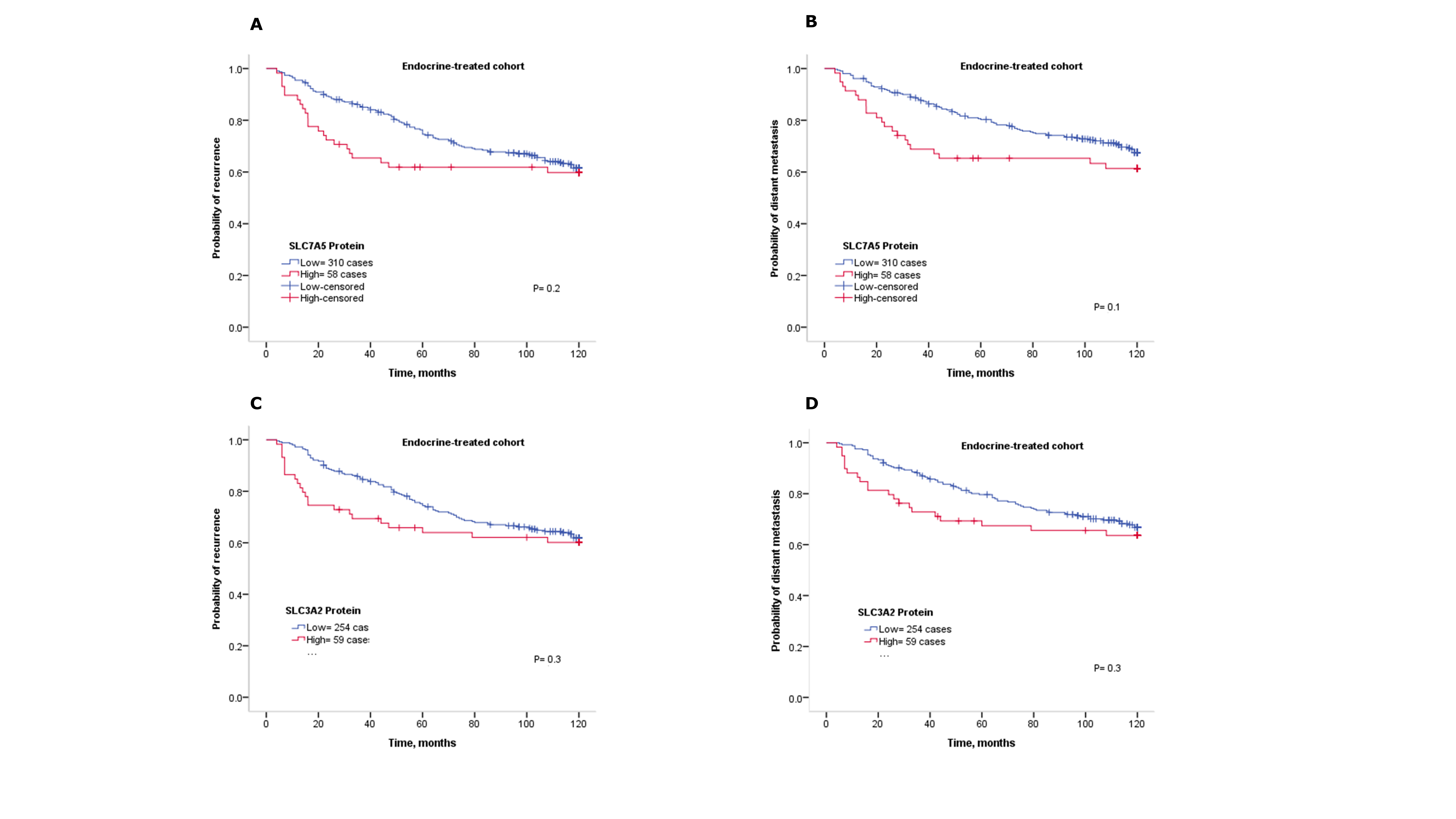

Supplement: Supplementary file 1 [file ijms-21-01407-s001.zip › ijms-707838-supplementary.tif]
